# ECG challenge: identifying a critical pattern in a patient with chest pain and pre-existing right bundle branch block

**DOI:** 10.1093/ehjcr/ytaf192

**Published:** 2025-04-12

**Authors:** Miguel Ferrer-Menéndez, Carlos González-Freixa, Meritxell Santaló-Corcoy

**Affiliations:** Department of Cardiology, Hospital de la Santa Creu i Sant Pau, Universitat Autònoma de Barcelona, Sant Quinti, 89, Barcelona 08025, Spain; Department of Cardiology, Hospital de la Santa Creu i Sant Pau, Universitat Autònoma de Barcelona, Sant Quinti, 89, Barcelona 08025, Spain; Department of Cardiology, Hospital de la Santa Creu i Sant Pau, Universitat Autònoma de Barcelona, Sant Quinti, 89, Barcelona 08025, Spain

A 58-year-old man with a history of hypertension, dyslipidaemia, and a known complete right bundle branch block (RBBB) presented to the emergency department with persistent chest pain at rest for 1 h. The pain radiated to his left arm and was accompanied by diaphoresis and nausea.

On examination, he was haemodynamically stable, euvolaemic, and well-perfused. Lung auscultation was clear, with no added sounds or crepitations. Heart sounds were rhythmic and normal, with no murmurs or pericardial rub.

A 12-lead electrocardiogram (ECG) was obtained and is shown below.

**Figure:**
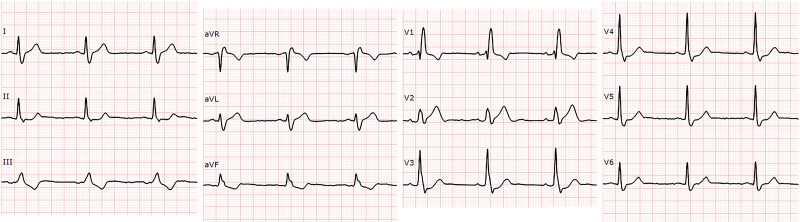


## Question 1

Which of the following is NOT a characteristic finding in complete RBBB?

- Normal R wave peak time in leads V5 and V6
- RSR′ in leads V1 and V2
- S wave duration greater than R wave duration in leads I and V6, or an S wave > 40 ms
- Upright T waves in the right precordial leads and inverted T waves in the left precordial leads
- R wave peak time > 50 ms in lead V1

Correct answer: D.

## Discussion and explanation

In RBBB, T waves are typically discordant with the QRS complex, leading to inverted T waves in the right precordial leads and upright T waves in the left precordial leads. However, in this ECG, lead V2 shows a concordant upright T wave, which is atypical for isolated RBBB and may indicate an underlying pathological process.^1^ All other options describe classic RBBB findings.

## Question 2

What is the next appropriate step in management?

- Serial troponins at 0/1 h or 0/2 h and clinical decision based on results
- Emergent coronary angiography
- Load dual antiplatelet therapy
- Initiate anticoagulation and perform CT angiography to rule out pulmonary embolism
- Perform ischaemia testing to guide subsequent catheterization

Correct answer: B.

## Discussion and explanation

The patient presents with persistent angina and an ECG showing ST elevation in leads I, aVL, and V2, along with reciprocal ST depression in the inferior leads, consistent with an acute ST-elevation myocardial infarction. Emergent coronary angiography is the most appropriate next step. While pre-treatment with aspirin and a P2Y12 inhibitor may be considered, it carries a class IIb recommendation in the 2023 ESC guidelines for acute coronary syndromes due to limited supporting evidence.^2^ Alternative options would lead to diagnostic delays or inappropriate management.

## Question 3

Which coronary artery is most likely responsible for the infarction?

- Diagonal branch of the left anterior descending artery (LAD)
- Septal branch of the LAD
- Obtuse marginal branch of the circumflex artery
- Posterior interventricular artery
- Posterolateral branch

Correct answer: A.

## Discussion and explanation

The ECG shows a pre-existing RBBB with new ST elevation in leads I, aVL, and V2, along with a concordant upright T wave in V2 and reciprocal ST depression in lead III. This pattern, known as the *South African Flag Sign*, indicates a high lateral infarction, typically involving a diagonal branch (see Supplementary material online, *Figure S1*).^3^ Coronary angiography confirmed a 100% occlusion of the second diagonal branch (see Supplementary material online, *Video S1*). South African Flag Sign is rarely reported in RBBB patients, making this case notable. Recognizing this pattern is crucial for early intervention and improved patient outcomes.

## Supplementary Material

ytaf192_Supplementary_Data

## Data Availability

The data that support the findings of this study are available from the authors upon request.
