# Supplementary figures and images for: ECG challenge: identifying a critical pattern in a patient with chest pain and pre-existing right bundle branch block
Source: Eur Heart J Case Rep. 2025 Apr 12;9(4):ytaf192. doi: 10.1093/ehjcr/ytaf192 (PMC12022474; doi:10.1093/ehjcr/ytaf192)

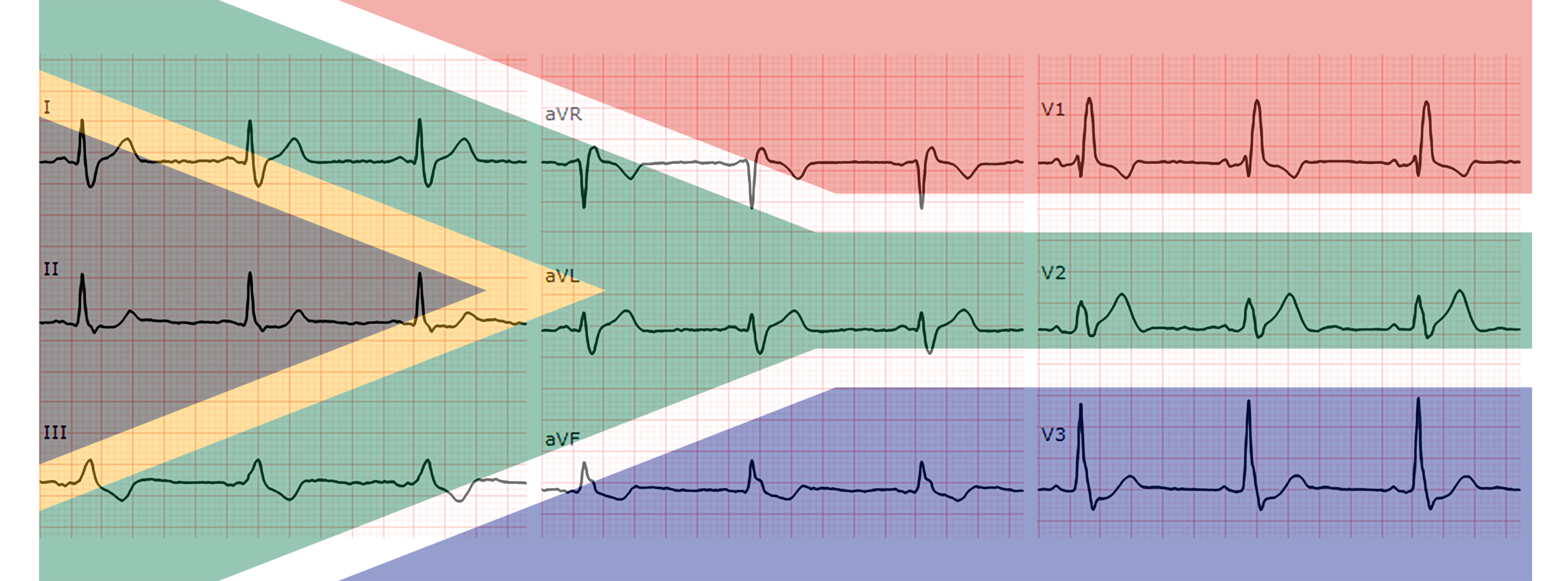

Supplement: ytaf192_Supplementary_Data [file ytaf192_supplementary_data.zip › Figure S1.jpg]
